# Benchmarking p*K*_a_ Prediction Algorithms against an Extensive, Public Data Set

**DOI:** 10.1021/acs.jcim.6c00107

**Published:** 2026-04-06

**Authors:** Levente Sipos-Szabó, Dávid Bajusz, György T. Balogh, György M. Keserű

**Affiliations:** † Medicinal Chemistry Research Group and Drug Innovation Centre, 280964 HUN-REN Research Centre for Natural Sciences, Magyar tudósok krt. 2, 1117 Budapest, Hungary; ‡ Department of Organic Chemistry and Technology, Faculty of Chemical Technology and Biotechnology, Budapest University of Technology and Economics, Műegyetem rkp. 3, H-1111 Budapest, Hungary; § Department of Pharmaceutical Chemistry, Semmelweis University, Hőgyes Endre u. 9, 1092 Budapest, Hungary; ∥ Center for Pharmacology and Drug Research & Development, Semmelweis University, Üllői út 28, 1085 Budapest, Hungary; ⊥ Department of Chemical and Environmental Process Engineering, Faculty of Chemical Technology and Biotechnology, 61810 Budapest University of Technology and Economics, Műegyetem rkp. 3, H-1111 Budapest, Hungary

## Abstract

Accurate prediction of proton dissociation constants (p*K*_a_) is essential for downstream drug discovery and molecular modeling workflows. While several proprietary p*K*_a_ prediction tools have been established as popular choices in the field, open-source solutions provide viable alternatives for large-scale computational workflows. In particular, machine learning approaches have recently emerged as a promising orthogonal route to traditional empirical methods. However, many of these algorithms were benchmarked on small, disjoint data sets, with inconsistencies in p*K*_a_ data interpretation, particularly for polyprotic molecules. To address these challenges, we assembled a comprehensive data set of over 90,000 experimental aqueous p*K*_a_ values spanning over 31,000 unique molecules from scattered online resources, with each entry annotated for charge state transitions and microspecies distributions. This data set, made accessible through the pKahub online database, represents one of the largest publicly available collections of annotated p*K*_a_ data to date. We used this resource to benchmark seven p*K*_a_ prediction methods, including three commercial tools (ACD/Labs, Chemaxon, and Epik) and four open-source machine learning models (MolGpKa, pKaSolver, QupKake, and Uni-p*K*_a_).

## Graphical abstract

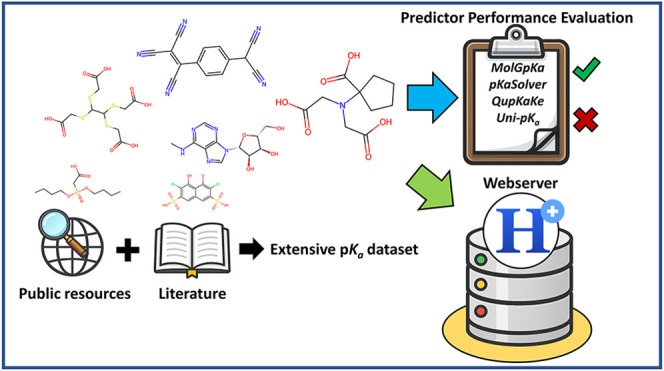

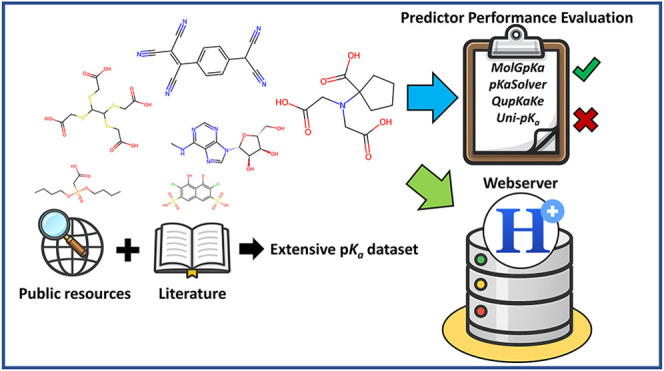

## Introduction

Many molecules tend to undergo spontaneous ionization in aqueous (or more generally, protic) media, mainly due to association or dissociation of mobile protons.^1^ According to the Bro̷nsted–Lowry acid–base theory, this behavior can be quantified by the reaction equilibrium constant of proton dissociation or association corrected by the concentration of the solvent. In practical scenarios, the negative logarithm is used for equilibrium constants; these are called p*K*_a_ and p*K*_b_ for acidic and basic reactions (proton dissociation and association), respectively. Ionization can have a drastic effect on physicochemical properties, such as solvation, lipophilicity, membrane permeability, and supramolecular interactions, all of them being crucially important aspects of drug efficacy and safety.^2,3^ Enumerating ionized states for small molecules is especially important for structure-based molecular modeling and virtual screening workflows. The protonation state of the ligand greatly affects the secondary interaction types and strengths with protein binding sites, such as electrostatic interactions, hydrogen bonding, and van der Waals interactions.^4^

Since large-scale proton dissociation constant determination experiments are infeasible for many, there are multiple methods for the computational prediction of p*K*_a_ values. (Crucially, these methods can also process virtual molecules not yet synthesized, where experimentation is, by definition, not available.) These can be theoretical approaches utilizing high-level energy calculations,^5,6^ such as density functional theory (DFT) along with thermodynamic cycles and advanced solvation models like COSMO^7^ or EC-RISM.^8,9^ Trading precision for higher throughput, empirical methods are based on QSPR approaches, using large, experimentally determined p*K*_a_ data sets to train prediction models on chemical features to determine the p*K*_a_ value of the query compound or functional group.^10,11^ For common use cases, empirical methods are far more utilized due to their computation time advantage, as ab initio or DFT-based methods can require a day’s worth of computation on computer clusters for a single molecule, while empirical methods usually take a few seconds or even less to evaluate a compound on a desktop computer.^6^ A perhaps less discussed advantage of empirical methods is that while they have a more limited applicability domain in accordance with their training data set, they usually outperform theoretical approaches in benchmarking studies.^12^ Therefore, empirical methods have been the established choices for general use cases such as ADMET property prediction or structure preparation for molecular modeling, with the most widely used alternatives being proprietary software, like Percepta from Advanced Chemistry Development, Inc. (ACD/Labs), and the property calculator modules of Chemaxon or Schrödinger Epik.^13^ This is especially the case for methodologies relying on linear free energy relationships (LFER), which were one of the leading paradigms in empirical p*K*_a_ prediction before the advent of machine learning approaches.^14,15^

In contrast, freely available and open-source applications for general use have been scarcer. This is especially the case for the full enumeration of possible protonation states for a molecule, which is required by advanced property predictions and downstream molecular modeling tasks such as virtual screening campaigns. To the best of our knowledge, the only mature tool for this task is Dimorphite-DL, which uses heuristic rules for enumerating the ionization states of predefined centers, according to a given pH.^16^ A freely available tool for the quantitative evaluation of ionized microspecies is still a great need. This is also the case for many commercial products, as they usually provide only microspecies distributions through graphical user interfaces, limiting the use cases for enumerating large compound libraries.

Recent years have seen the large-scale adaptation of machine learning methods.^17–19^ In machine learning, the model adapts to the chemical input data, which circumvents the need for the laborious, hand-crafted equations needed for previous methods such as LFER, making academic and open-source development more feasible. In the past few years, several new predictor models based on deep neural networks were released and many of these models are freely available and show competitive performance with commercial methods.^17,20,21^

The cornerstone of every machine learning method is the quality and amount of data used to train it.^22^ Compared with the training requirements of advanced deep learning methods, the available amount of experimental p*K*_a_ data is rather low. To handle this, almost every recently published method applies a pretraining-fine-tuning paradigm: in the pretraining phase, a large amount of calculated p*K*_a_ values are utilized from the ChEMBL database,^23^ which are then followed by fine-tuning on a smaller set of experimental data. For experimental p*K*_a_ data, the largest public data set is the iBonD databank,^24^ which contains over 40,000 p*K*_a_ values to date. Interestingly, there is no restricting license on these data, but the database can only be parsed in a purely visual manner, making it difficult to adapt for programmatic use cases. Large portions of this data set were manually collected and released as training sets in recently published predictors, making them more easily accessible.^25^ Other predictors used orthogonal training sets like the p*K*_a_ data set released with the Datawarrior^26^ software, or its modified versions.^27^ This means that most method developers utilize only a subset of the publicly available data, for example, the large and reliable IUPAC digitized *K*_a_ dataset is rarely used for training or benchmarking these new methods.^28^

The increasing impetus in method development has also highlighted some serious misconceptions concerning p*K*_a_ data interpretation in the scientific community, particularly with polyprotic small molecules.^29^ The main source of confusion seems to be the oversimplification of the more complex ensemble of the ionized microspecies of molecules with more than one ionization center. A common mistake arises from the conceptually tempting notion of assigning a single p*K*_a_ value to the whole molecule, or to a single functional group. This practice works for simple acids and bases but leads to inconsistencies when dealing with more complex molecules. This is also reinforced by the fact that almost every available experimental p*K*_a_ data point describes a macroscopic average of a given set of microspecies. For a comprehensive discussion of this topic, we would like to point the reader to the article of Fraczkiewicz.^30^ These problems also leaked into frequently used data sources such as the ChEMBL database and affect the quality of many predictors using these data, as pointed out by Zheng et al.^29^ To this end, it is recommended to clearly annotate the exact charge state transition (e.g., “0 to −1”), along with published p*K*_a_ values, an information that is lacking in public data sources. To date, there is also no widely accessible p*K*_a_ data repository that contains detailed microspecies data along with p*K*_a_ values. To address the above-mentioned issues, we assembled the largest data set of experimental aqueous p*K*_a_ values from the literature and various public sources to date, containing over 90,000 data points across more than 31,000 unique molecules. Each p*K*_a_ value was annotated with a charge state transition and relevant microspecies distributions. We make this data set accessible through a Web server called pKaHub (http://pkahub.ttk.hu). Here, we use this extensive data set to benchmark and compare three commercial and four open-source machine learning-based p*K*_a_ prediction methods, namely, ACD/Labs, Chemaxon, Epik,^13^ MolGpKa,^17^ pKaSolver,^31^ QupKake,^20^ and Uni-pK_a_.^21^

## Methods

### p*K*_a_ Terminology

The term p*K*_a_ denotes the negative base-10 logarithm of the equilibrium constant for a proton dissociation reaction expressed relative to the activity of the solvent. In the simplest case, it describes deprotonation of a conjugate acid at a single acidic functional group. More generally, for molecules with multiple ionizable sites, a deprotonation step occurs between two microspecies that differ in the presence of a single proton; the equilibrium constant for such a site-specific transition is described by a microscopic p*K*_a_ (micro-p*K*_a_). Most experimental approaches for determining proton-transfer equilibria rely on titration-based measurements, which typically report transitions between overall (net) charge states of the molecule. These observed transitions therefore correspond to averages over multiple underlying microscopic protonation/deprotonation events rather than a single site-specific process and are commonly referred to as macroscopic p*K*_a_ (macro-p*K*_a_) values.

### Predictors

We selected four open-source, deep learning-based p*K*_a_ predictors published in recent years as the basis of our evaluation. These methods have been reported to perform comparably to commercial tools on small benchmark data sets (e.g., SAMPL), while remaining readily accessible through publicly available code and pretrained weights. Together, they also reflect the recent evolution of deep-learning architectures for p*K*_a_ prediction. MolGpKa was among the earliest graph neural network (GNN)-based predictors for small organic molecules. It was trained primarily on calculated p*K*_a_ values (ACD/Labs) available through the ChEMBL v25 data set. pKaSolver was likewise trained on ChEMBL-derived p*K*_a_ values calculated with Epik, but it additionally introduces a fine-tuning stage by using experimentally measured p*K*_a_ data to improve predictive accuracy. pKaSolver uses Dimorphite-DL to enumerate one dominant microspecies for the different charge states of the query molecule and performs sequential ionization prediction by predicting the micro-p*K*_a_ between the enumerated forms; accordingly, we treat it as a microscopic p*K*_a_ predictor. For the Epik-derived version in the original work, the model weights could not be shared due to licensing restrictions. The open version (called pKaSolver-lite in the original article) of the model was trained using only the experimental data. As our goal was to track the performance of freely available methods, we did not attempt to recreate the proprietary variant. QupKake similarly includes an initial training phase on calculated p*K*_a_ values made accessible through ChEMBL, although in this case, the calculations were performed with Marvin (Chemaxon), rather than ACD/Labs, followed by experimental-data fine-tuning. A distinctive feature of QupKake is its use of quantum-chemical descriptors computed with the semiempirical GFN2-xTB method.^32^ Its protonation-site identification is also atypical: whereas most methods rely on predefined protonation templates, QupKake employs a separate neural network to detect ionizable sites. This model was trained using ionization-site scanning calculations for ChEMBL molecules generated with the CREST semiempirical quantum chemistry workflow.^33^ Finally, Uni-p*K*_a_ follows a different strategy. For a given query molecule, it enumerates possible microspecies using a template-based set of ionization rules to identify candidate sites, generating states through sequential protonation and deprotonation. For each microspecies, it predicts a dimensionless free energy from which macroscopic and microscopic p*K*_a_ values can be derived via Boltzmann averaging. Uni-p*K*_a_ also uses ChEMBL-based training and experimental-data fine-tuning, primarily drawing experimental labels from the DataWarrior and Novartis data sets. We evaluated Uni-p*K*_a_ in two configurations. One with the simplified template with around 30 site definitions, as recommended by the authors for general use, and the other with the full template compiled by the authors, which includes many ionization sites that are rarely relevant for typical drug-like molecules. Although the weights we used differ from those reported in the original publication, this implementation remains the most accessible in terms of practical utility. The p*K*_a_ predictors used in this study are summarized in Table 1.

**Table 1:** Open-Source and Commercial p*K*_a_ Predictors Included in the Benchmarks

**name**	**proprietary or open-source**	**predicted p** *K*_**a**_ **type (with batch calculations)**	**predictor architecture**
MolGpKa^17^	open-source	micro	graph neural network
pKaSolver^31^	open-source	micro with sequential ionization	graph neural network
QupKake^20^	open-source	micro	graph neural network
Uni-p*K*_a_ with simple template^21^	open-source	micro and macro	SE(3)-transformer-based foundational model
Uni-p*K*_a_ with full template^21^	open-source	micro and macro	SE(3)-transformer-based foundational model
ACD/Labs Classic^34^	proprietary	macro	LFER
ACD/Labs GALAS^34^	proprietary	macro	undisclosed
Chemaxon^35^	proprietary	micro and macro	undisclosed
Epik^13,36^	proprietary	macro	graph neural network

In the following sections, we generally refer to MolGpKa, pKaSolver, QupKake, and Uni-p*K*_a_ as open-source, and Chemaxon, Epik, and ACD/Labs as commercial or proprietary predictors. MolGpKa, pKaSolver, and QupKake were implemented using the code and weights distributed on GitHub, with minimal modifications in some cases to carry out batch calculations. QupKake was used as the command-line utility. Uni-p*K*_a_ was used according to the code on Bohrium with the uploaded weights (www.bohrium.com/notebooks/38543442597). The Uni-p*K*_a_ code for microstate enumeration was changed to enumerate states in the −6 to +6 charge window. Epik was used in the Schrodinger 2025-2 package (epikx), and input was processed using Ligprep before predictions with enumerated charge states set to −6 to +6. The same Epik workflow was used for the creation of the database and for performance evaluations. Chemaxon p*K*_a_ calculation was carried out using the cxcalc command-line utility with the “large model” selected.

### Data Set Collection and Preparation

We collected various p*K*_a_ data compilations from the literature and public data resources. Many literature data sets already had citations in other works and somewhat-established names, being mostly referred to by the name of the first author of the original publication. Here, we kept this convention for unnamed, lesser-known data sets. As an exception, the name “AvLiLuMoVe” refers to the filename used in the original publication’s Supporting Information, where it is labeled the “Literature” data set (we avoided this label here, as it could be misleading in our context). In the text, we refer to these as “source data sets”.

The literature compilations included the Settimo, Hunt, Jensen, Caine, Manchester, Organic Oxygen Acids, and Nitrogen Bases data sets, which were acquired from the Supporting Information of these articles. Chemical structures and p*K*_a_ values from the Caine and Manchester data sets were digitized by us using the OSRA optical recognition software.^37^ The Baltruschat ChEMBL data consisted of experimental proton dissociation constant data mined by Baltruschat et al. from ChEMBL. The AvLiLuMoVe and Novartis data sets were also acquired from this source. The Datawarrior data set was downloaded from the databanks of the Datawarrior software. The IUPAC digitized p*K*_a_ dataset is a combined effort to digitalize the p*K*_a_ data from various chemistry handbooks. The AttenGpKa training set is a large compilation of data which was reportedly acquired via the manual mining of the iBonD database.^24^ The OCHEM data was acquired from the Online Chemical Modeling Environment website, a public data repository. The QSARToolbox data was downloaded from the “pKa” and “pKa OASIS” database end points of the QSARToolbox software. The SAMPL data sets were acquired from the associated GitHub repositories of the challenges. The collected data sets are summarized in Table 2.

**Table 2:** Summary of the Collected Source Datasets before and after Data set Processing, with the Number of Contained p*K*_a_ and Other Types of Dissociation Constant Values and the Number of Unique Molecules (Unique at the Dataset Level)

	**before processing**		**after processing**	
**source data set name**	**proton dissociation constants**	**unique molecules**	**p** *K*_**a**_ **entries**	**unique molecules**
Settimo^38^	622	426	622	426
Hunt^39^	2435	2242	2430	2237
Jensen^40^	53	48	53	48
Caine^41^	78	71	78	71
Manchester^42^	142	85	142	85
Organic Oxygen Acids and Nitrogen Bases^43^	1143	1123	1143	1123
Baltruschat ChEMBL^44^	8106	6303	7752	6093
AvLiLuMoVe^44^	123	123	123	123
Novartis^44^	280	280	280	280
Datawarrior^26^	7913	6375	7495	6050
IUPAC digitized p*K*_a_ dataset^28^	24,211	10,615	18,513	8180
AttenGpKa training set^25^	26,522	15,712	13,556	10,384
OCHEM^45^	21,687	11,855	20,708	11,112
QSARToolbox^46^	28,926	13,390	24,609	12,341
SAMPL6 ^47^	31	24	31	24
SAMPL7 ^48^	20	20	20	20
SAMPL8 ^49^	24	21	24	21
euroSAMPL1 ^12^	35	35	35	35
combined	122,351	38,701	97,614	31,250

Individual source data sets were processed separately. For the processing, we included only p*K*_a_ or p*K*_b_ type of proton dissociation or association constants and converted all p*K*_b_ values to p*K*_a_ by subtracting them from 14.0 (p*K*_w_, from the self-dissociation constant of water). We excluded entries where the chemical structure could not be processed by RDKit, except in a few cases when we manually repaired structures with a problematic SMILES representation regarding the aromaticity of indole rings.

Entries were also excluded if there was an implication that the measurement was not carried out in water. This included entries from the AttenGpKa training set and the IUPAC digitized p*K*_a_ data set, where we excluded all entries where the cosolvent content was greater than 5% or when the cosolvent content was not quantitatively specified. Entries in the IUPAC digitized data set with nonconventional experimental settings, such as covalent hydration, excited states, or extreme pressure, were also filtered out, as well as entries where the remark specified a lack of information on the experimental conditions.

Charge state transition assignment was carried out for every experimental p*K*_a_ value by matching them to the calculated macroscopic p*K*_a_ values by Epik. By manual examination of the Jensen, Manchester, and all SAMPL data sets, we could assume that in cases of multiple, different p*K*_a_ entries for the same molecule, the entries refer to different charge state transitions. For these values, we used an order-preserving matching algorithm (Figure 2), which matches the order of the experimental and predicted p*K*_a_ values when ordering them in an ascending (or descending) order. The AvLiLuMoVe and Novartis data sets contained only one p*K*_a_ value for a single molecule: in this case, the charge state transition with the lowest difference to the predicted value was chosen. For the Settimo, Hunt, Caine, Organic Oxygen Acids and Nitrogen Bases, AttenGpKa, OCHEM, QSARToolbox, Baltruschat CheMBL, and DataWarrior data sets, it was not certain whether different values belong to different transitions for the same molecule. In this case, a given experimental value was matched to the predicted value that was closest to it. For the IUPAC digitized p*K*_a_ dataset, there is a “pKa type” label associated with every entry. This describes the type of ionization constant the numeric value refers to, such as “pKb” or “pKa”, and in some cases, also describes whether the constant refers to a different dissociation step (e.g., “pKa1” and “pKa2”) for polyprotic molecules. This proved to be consistent among a large section of the data set. However, in a few hundred cases, we found the notations to be somewhat inconsistent. Therefore, for every unique molecule in the data set, we grouped the p*K*_a_ values by their types and checked (i) if there were any two values within one group with an absolute difference greater than 1.0, or (ii) if there were any pair of values between two groups with an absolute difference smaller than 0.5. When the groupings passed the check, we assigned the experimental p*K*_a_ values with order-preserving matching.

We excluded all data where the structure could not be processed by Epik or where we could not get any predictions. This usually involved cases with an erroneous structure or structures with more exotic ionization centers, such as aliphatic or aromatic C–H deprotonation, except for 8 values from the Novartis data set and two values from the Hunt data set which were processed manually. To filter out possibly misannotated or erroneous experimental values from further processing, we excluded all molecules and associated p*K*_a_ entries in a given data set, where there was at least one matched experimental vs predicted p*K*_a_ value pair with a greater difference than 4.

We also extracted temperature and ionic strength values for each data point where possible, although temperature values were only specified for some of the IUPAC and OCHEM entries. Ionic strength values were only provided by the IUPAC digitized p*K*_a_ dataset for a subset of entries.

### Averaging p*K*_a_ Values for Charge State Transitions

We define an aggregated set of experimental p*K*_a_ values for every unique molecule in the full database by averaging every experimental p*K*_a_ value assigned to the same charge state transition of that molecule (from now on, we refer to these values as the experimental p*K*_a_ values of that molecule for that transition). Recognizing that several p*K*_a_ values across different source data sets could be originating from the same measurement, we applied a cross-data set deduplication procedure before this averaging step. For each molecule, p*K*_a_ values are grouped by source data set and charge state transition. If the same numeric p*K*_a_ value appears for the same transition in multiple source data sets, it is likely a duplicate entry propagated across source data sets, so it is counted only as many times as the minimum occurrence count in any of those source data sets. (If a value appears in only one source data set, its count is kept as-is.) The resulting counts determine how many times each p*K*_a_ value enters the final list and contributes to the aggregated p*K*_a_ for the given charge state transition during averaging.

### Benchmark Data Sets for Predictor Evaluation

We compiled three benchmark data sets from the combined database of 31,250 molecules to test the open-source predictors against the commercial ones by applying a sequence of filtering steps and grouping the remaining molecules. The process for creating the three data sets is shown in Figure 1.

**Figure 1:**
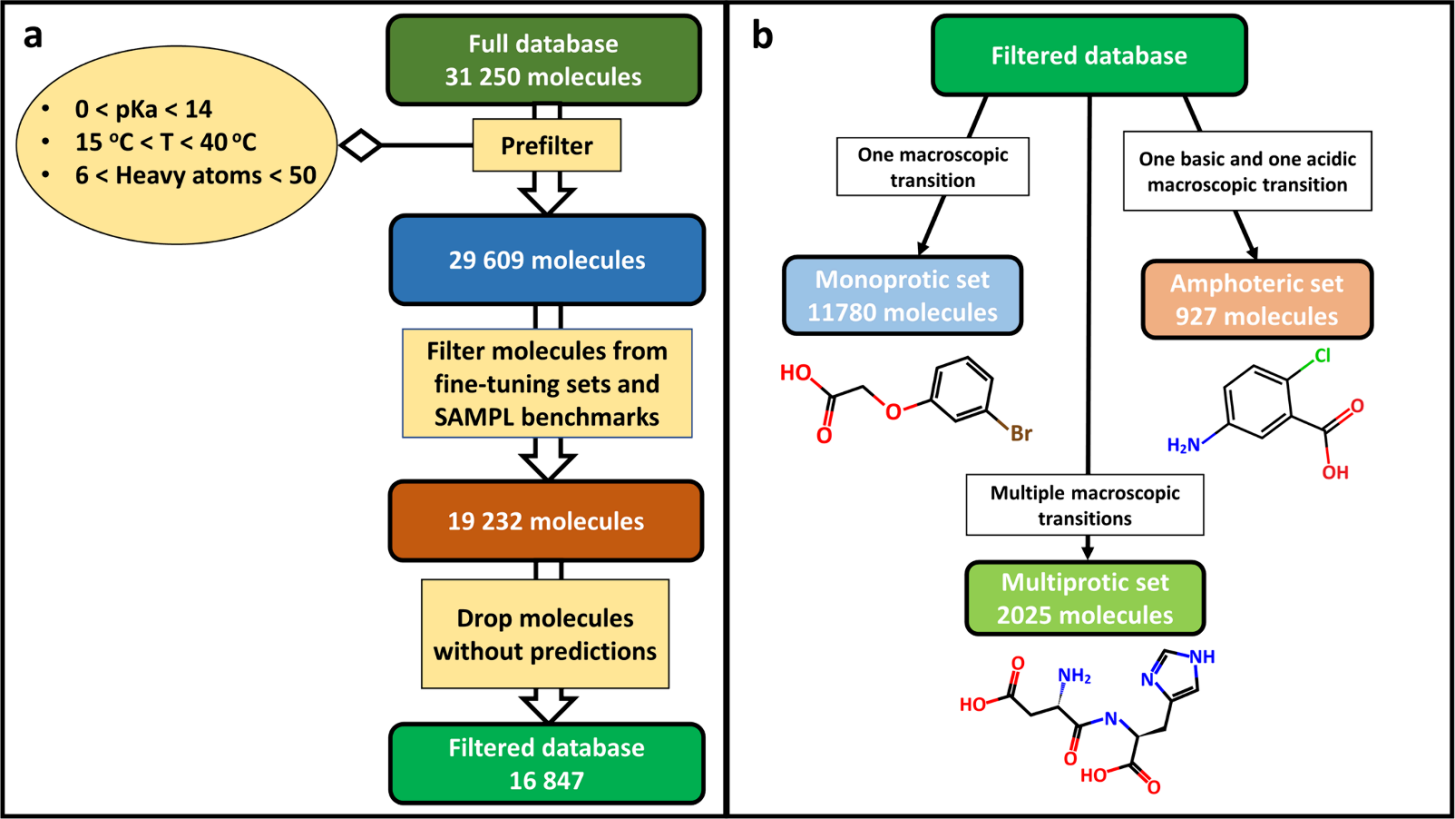
(a) Filtering steps applied to the full database. (b) Three benchmark data sets for predictor validation were compiled from the filtered and curated database.

We included only molecules with all of their p*K*_a_ values between 0 and 14. We filtered out every molecule that has a heavy atom count lower than 6 or greater than 50. We also filtered every p*K*_a_ entry which had an assigned temperature value outside of the 15–40 °C interval. This reduced the number of molecules to 29,609. From this, we filtered out every molecule which was also present in the fine-tuning data of any of the open-source predictors or was in any of the popular benchmark sets, such as the SAMPL sets or the Novartis set. (The latter was also excluded because it was also a part of the Uni-p*K*_a_ fine-tuning set for the used weights.) This filtering left 19,232 molecules in the set. Stereochemistry was omitted when these comparisons were made with the fine-tune set, as some predictors do not consider stereocenters in their predictions and in some cases, we found the handling of stereochemistry information inconsistent in these data sets. To test the predictors on equal footing using the same set of molecules, we filtered out any molecule for which at least one of the relevant predictors did not yield a valid prediction. A prediction was considered sufficient if the number of predicted p*K*_a_ values was at least the number of experimental p*K*_a_ values available for that molecule. We defined three groups of molecules. Molecules with a single experimental p*K*_a_ and an assigned charge-state transition of 0 to −1 (acid) or 1 to 0 (base) were labeled as monoprotic. Molecules with exactly two experimental p*K*_a_ values and assigned charge-state transitions of 0 to −1 and 1 to 0 formed the amphoteric group. Molecules with more than one experimental p*K*_a_ value that were not labeled as amphoteric were labeled as polyprotic. For the monoprotic and amphoteric groups, we excluded any molecule with an insufficient prediction from any predictors. For the polyprotic group, only macroscopic predictors were considered. After filtering, the final data set comprised 16,847 molecules. The filtered molecules were compiled into the monoprotic, amphoteric, and polyprotic data sets according to their labels. We did not include polyprotic molecules in the benchmarking data sets, which had only one assigned transition where both involved states had a nonzero net charge, as it implies that the molecule has experimentally undetected charge state transitions.

### Cheminformatics Tools

In the majority of cases, RDKit (version 2024.03.5) was used for the processing of chemical data. For molecular similarity calculations, Morgan fingerprints of radius 4 with 2048 bits were generated, and similarity was calculated using the Tanimoto index.^50^ In the evaluations, two molecules were considered equivalent if they had the same InChI strings. Data set processing and the evaluation of predictors were performed using in-house Python scripts. Similarity between two sets of molecules was calculated by averaging the similarity values of all of the molecules in the smaller set with their most similar counterpart from the partner set.

### One-Way ANOVA

To establish statistical significance between the distributions of absolute prediction errors among the evaluated micro- and macro-p*K*_a_ predictors, one-way analysis of variance (ANOVA) was performed using the scipy (version 1.16.3) Python package. Absolute errors between experimental and predicted p*K*_a_ values were aggregated across all molecular benchmark data sets (monoprotic, amphoteric, and polyprotic benchmark sets) for each predictor, yielding one independent sample per predictor. The null hypothesis that all predictors share a common population mean absolute error was tested at a significance level of α = 0.05. Upon rejection of the null hypothesis, pairwise post hoc comparisons were conducted to identify which specific predictor pairs differed significantly. Five established parametric post hoc tests were applied: Scheffé’s test, Tamhane’s T2 test, the pairwise Student’s *t-*test with Bonferroni-Holm correction, Tukey’s range test, and Tukey’s honestly significant difference (HSD) test. All post hoc procedures were implemented using the scikit-posthocs library (version 0.12.0) and applied at the same α = 0.05 threshold. To obtain a single, robust significance determination for each predictor pair, the binary outcomes of the five post hoc tests were aggregated by majority vote: a pair was deemed statistically significantly different if at least three of the five tests independently reported *p* < 0.05 for that pair.

### Web Server

The pKaHub web server was implemented using the Django (version 5.2) framework with the SQLite database engine. The microspecies distribution for a given charge state of a molecule is also provided by using the microstate enumerator of Uni-p*K*_a_. Here, we primarily used simple templates for generating microspecies; in cases when the enumeration with simple templates was insufficient to provide any microspecies for the given charge state, full template enumeration was used. Microspecies in a given charge state were filtered by discarding every structure with a state population of less than 5% according to the predicted free energy by Uni-p*K*_a_.

## Results and Discussion

### Assignment of p*K*_a_ Values to Calculated Charge State Transitions

A typical p*K*_a_ determination involves titrating the solution of the molecule across a defined pH range while continuously monitoring changes in a suitable physicochemical signal, most commonly via potentiometric or spectrophotometric measurements. In ideal cases, the observed signal can be described as a population-weighted sum of contributions from the relevant protonation states, provided that the individual states exhibit sufficiently distinct responses. (This assumption does not always hold, e.g., in spectrophotometric titrations of molecules lacking an adequate chromophore, different protonation states may produce indistinguishable spectra.) By fitting the pH dependence of this signal with an appropriate equilibrium model, one can extract the equilibrium constants (p*K*_a_ values) describing the system’s pH-dependent state transitions. In this setting, the measurement yields only a sequence of apparent transition values consistent with the measured pH range but does not directly identify the specific protonation states involved in each transition.

Assigning specific microscopic states to experimentally derived macroscopic constants is generally not straightforward, especially by experiment. A practical route to a more detailed description of the underlying transitions is, therefore, to align the experimental values with computationally predicted dissociation constants. In structure-based prediction frameworks, each predicted p*K*_a_ is associated with a defined protonation or microstate transition, providing the state-level annotation that is typically missing from experiment.

Formally, this amounts to matching two ordered sequences: an experimental set of length *N* and a predicted set of length *M*, where the goal is to assign each experimental value to one of the predicted values. We generally expect *M* ≥ *N*; if fewer transitions are predicted than observed, then the predictor has likely failed to enumerate the relevant ionization states. To obtain a fair and chemically consistent alignment, it is recommended that the matching algorithm should satisfy two principles.^51,52^ First, it should be order-preserving: if experimental values *e*_*i*_ and *e*_*j*_ (with *i* < *j*) are matched to predicted values *p*_*k*_ and *p*_*l*_, then the indices must satisfy *k* < *l*, preventing “crossing” assignments and preserving the monotonic progression of transitions with pH. Second, among all order-preserving matchings, the method should minimize the sum of absolute deviations between the matched values, $\overset{N}{\underset{1}{\sum }}\left|e_{n}-p_{m}\right|$ . These principles ensure numerically precise correspondences while respecting the relative magnitudes of the predicted transitions.

Notably, these principles do not require the matched predicted values to form a consecutive block within the predicted sequence. Allowing gaps allows the possibility of experimentally undetected transitions, leaving some predicted p*K*_a_ values unassigned. This choice is deliberately agnostic with respect to experimental sensitivity and imposes minimal constraints on the alignment problem. However, it implicitly assumes that experimentally undetected transitions are more common than large predictor errors. If, instead, one assumes that the experiment captures all transitions within the relevant pH window, then the matched predicted values should be consecutive; in that regime, the assignment reduces to a sliding-window alignment of the *N* experimental values against all length-*N* consecutive segments of the *M-*predicted values. We illustrate the two kinds of assignment paradigms for an example molecule in the data set on Figure 2.

**Figure 2:**
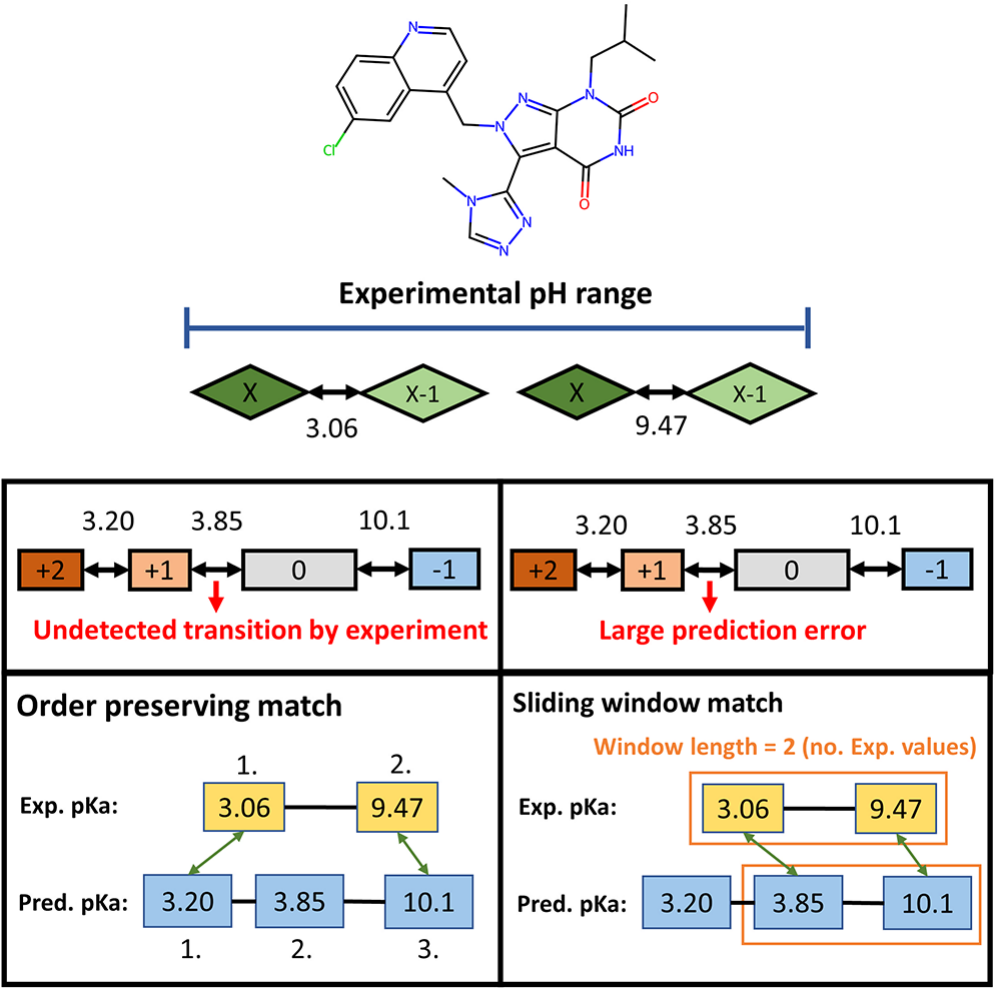
Assignment of experimental p*K*_a_ values to macroscopic charge state transitions through matching the numeric values of the experimental and calculated p*K*_a_s. Boxes refer to relevant (nonexotic) macroscopic charge states within a given pH range, and bidirectional arrows refer to macroscopic charge state transitions, with the assigned numbers referring to macro-p*K*_a_ values. When using order-preserving matching of the p*K*_a_ values, we assume that there could be “gaps” in the detected experimental state transitions. If we accept that the predictor could have large errors and the experiment registers all relevant macroscopic transitions, we can use a window search technique by enforcing a one-to-one correspondence in a consecutive order between the experimental and predicted values.

The problem becomes more challenging in large aggregated data sets, where metadata rarely indicate whether two reported p*K*_a_ values originate from the same experiment, or whether they refer to distinct experimental state transitions; in many cases, a p*K*_a_ value only has a single assigned chemical structure without sufficient labels. In this case, a maximally permissive strategy is to assign each experimental value to its closest predicted value. Under a stricter “complete observation” assumption, one would instead minimize the total assignment error subject to the consecutiveness of the selected predicted values, potentially allowing multiple experimental measurements to map to the same predicted transition.

As our compiled database spans a wide range of experimental methodologies and, possibly, a wide range of conditions, it is difficult to quantify the relative frequency of experimentally undetected transitions compared to the intrinsic prediction error of Epik, our chosen reference predictor method. Therefore, we apply the more agnostic order-preserving matching method, as this enforces the least amount of constraint and provides the least bias on our end.

### Combined Database Composition

The combined database consisted of 97,614 experimental p*K*_a_ values from 31,250 molecules after processing. The p*K*_a_ values that were assigned to the same charge state transition of the same molecule were averaged: we assigned these averaged p*K*_a_ values as the given molecule’s p*K*_a_ value for that given charge state transition (values with experimental temperatures outside of the 15–40 °C temperature window were excluded from the average). To filter duplicate data points between different data sets, we excluded any numerically equivalent p*K*_a_ values from different data sets within the same charge state transition, as described in Methods. With this method, we arrived at 37,654 distinct p*K*_a_ values for the molecules in the combined data set (including p*K*_a_ values with excluded temperature ranges, which extends this set with only 44 new p*K*_a_ averages). The distributions of averaged p*K*_a_ values, average number of experimental p*K*_a_’s assigned to a charge state transition, molecular weights, and charge state transitions, as well as the similarity of the different subdata sets (t-SNE plot) are shown in Figure 3.

**Figure 3:**
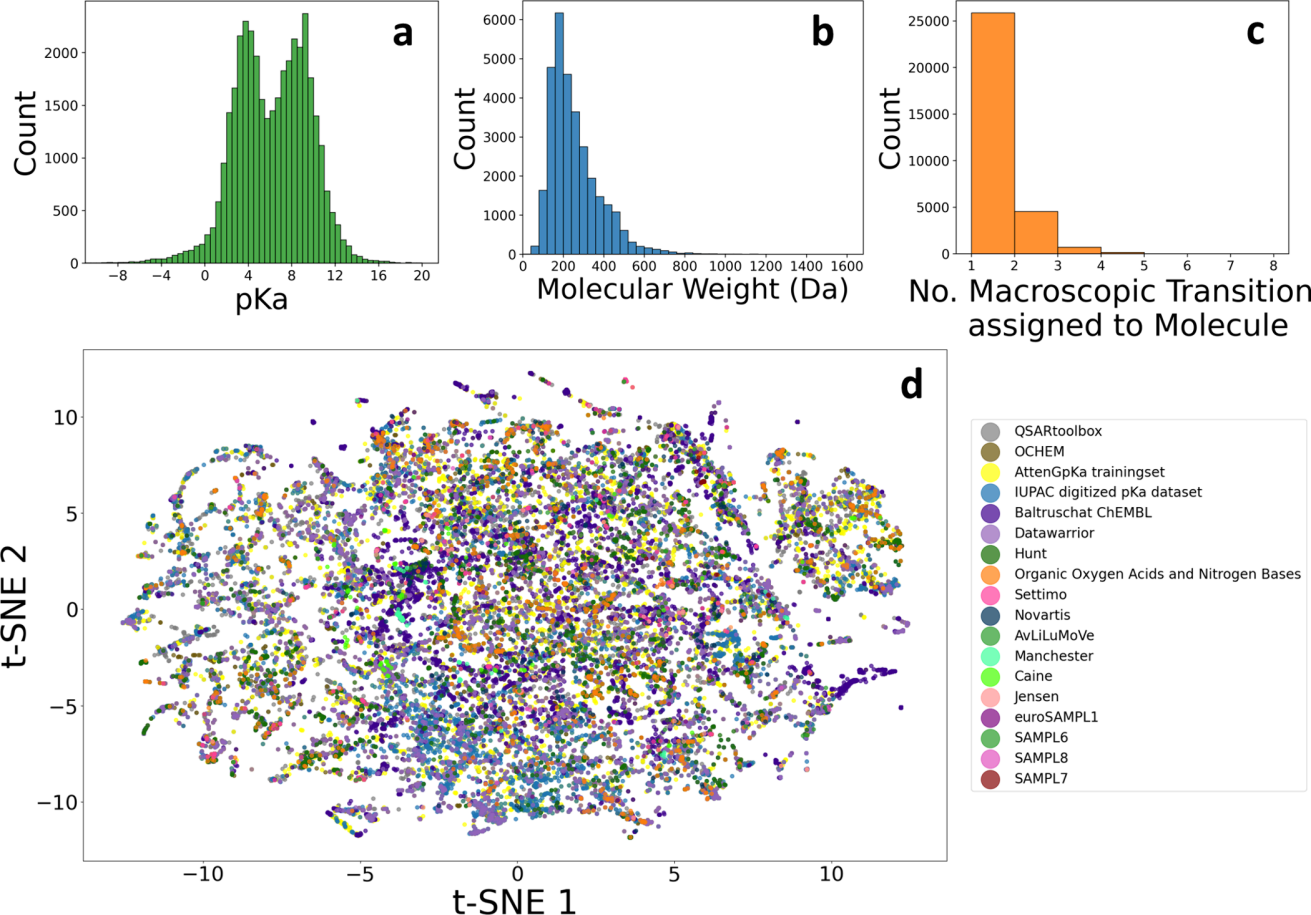
Distribution of (a) p*K*_a_ values in the combined database. (Averaged per charge state transition for a molecule, p*K*_a_ values with experimental temperatures outside the 15–40 °C temperature window were excluded from the average, numerically equivalent p*K*_a_ values from different source data sets were excluded.) (b) Molecular weights. (c) Number of charge state transitions assigned to a molecule. (d) t-SNE plot representing the chemical space of the source data sets.

The combined p*K*_a_ database aggregates values from diverse literature sources, yet experimental method annotations are frequently absent or nonstandardized. Consequently, deviations observed among replicate p*K*_a_ values for the same molecule and charge-state transition may reflect a mixture of true measurement variability and intermethod differences. To assess this, we quantified two complementary dispersion measures for transitions with at least two experimental entries: (i) the standard deviation and (ii) the largest pairwise difference in p*K*_a_ values. The range metric is particularly informative because it directly captures worst-case disagreement and remains interpretable, even for small replicate counts. Finally, to contextualize these results, we repeated the same analyses on the IUPAC digitized p*K*_a_ dataset, which includes explicit experimental method labels, allowing us to compare overall dispersion with method-stratified dispersion and thereby estimate how much of the observed spread is attributable to method heterogeneity versus within-method reproducibility. The distributions are found in Supplementary Figure S2. The mean standard deviation and largest pairwise difference are 0.20 and 0.38, respectively, with larger values quickly trailing off. These values are consistent with reported interexperiment deviations in other benchmarking studies,^38,44^ and are reasonably robust within a range that is sufficiently narrower than the deviation of predicted values vs experimental values.

To assess how the different source data sets overlap in their molecule content, we checked the number of unique molecules that only appear in the given data set and calculated the fraction of these molecules compared to the size of the source data set. The full report on this can be found in Supplementary Table S1. The most unique source data sets (by fraction of data set-specific molecules) proved to be the SAMPL, Novartis, Caine, and Manchester data sets. This is consistent with the notion that SAMPL sets and the Novartis set are used as benchmarking sets, and we can see that the digitization of the Caine and Manchester data set provided many unique molecules, even if the overall size of the data sets is small. The least unique source data sets were the Datawarrior, Organic Oxygen Acids and Nitrogen Bases, and OCHEM data sets. Interestingly, the SAMPL7 data set also proved to contain quite few unique molecules (15%), considering that this data set was used as the base of a competition between different p*K*_a_ predictors.

We provide a characterization of the distribution of acidic and basic structural moieties based on SMARTS pattern matching and the elemental composition of the molecules in the database in Figure 4. The database has a high prevalence of amines, basic aromatic nitrogens, and carboxylic acids and contains more molecules with only basic titratable groups than molecules with only acidic groups, but molecules with both basic and acidic groups are the most numerous (numerical data are provided in Table S2 by specific titratable groups—some of these are shown under umbrella terms like “carbon acids” in Figure 4). These trends reflect the overall composition of chemical spaces that are investigated in drug discovery.

**Figure 4:**
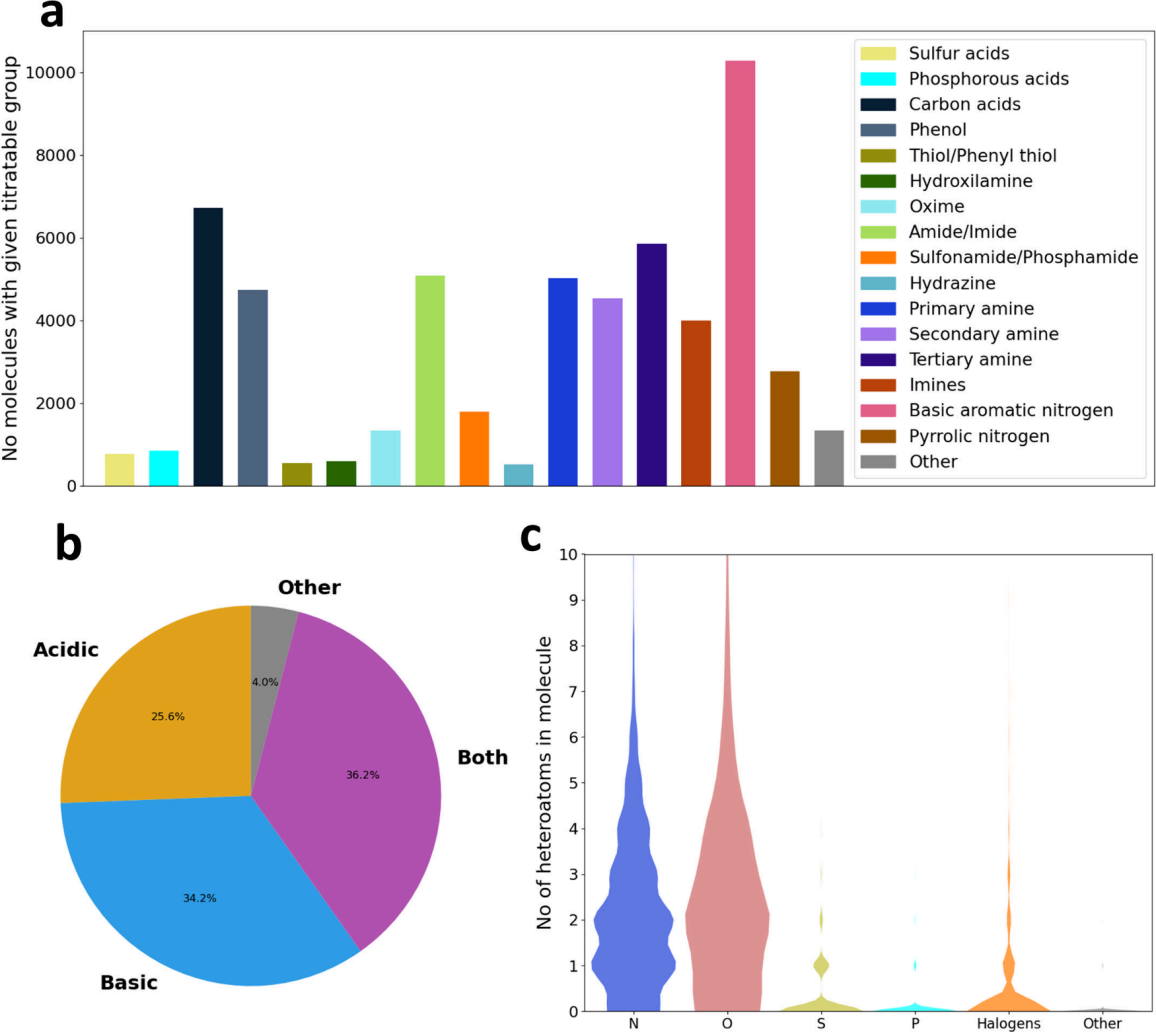
Distributions of (a) the number of molecules in the database that contain at least one titratable group in the given categories; (b) molecules with only acidic, basic, or both kinds of titratable groups (“Other” denotes molecules with only nonconventional titratable groups such as C–H acids). (c) Violin plot showing the distribution of the number of heteroatoms per molecule in the database.

### Evaluation of Commercial and Open-Source Predictors

For benchmarking recently developed prediction methods, the SAMPL and Novartis data sets have become widely used validation sets. The SAMPL data sets are particularly suitable because they were designed and carefully curated specifically to evaluate predictor performance. The Novartis data set originates from the work of Baltruschat et al.,^44^ who obtained the measurements through an industry collaboration with Novartis. We note that this data set is frequently misattributed to Liao et al.,^54^ likely because the original article itself appears to include an incorrect citation to the mentioned paper, instead of Novartis when citing this data set. Important limitations of these benchmarks are their size (few hundred molecules) and their composition, which tends to avoid more complex, polyprotic molecules. As noted previously, this can restrict conclusions about performance on chemically challenging systems.^53^ To evaluate open-source predictors against commercial reference methods under increasingly demanding conditions, we therefore compiled three benchmark data sets of increasing difficulty: a monoprotic data set containing molecules with a single reported charge-state transition, an amphoteric data set containing molecules with two transitions between the exact charge states of −1, 0, and +1, and a polyprotic data set consisting exclusively of molecules with a more complex set of charge state transitions. It is generally inadvisable to evaluate proprietary predictors on literature-derived data sets, because potential overlap between a model’s training data and the validation set cannot be assessed and may bias the results. Accordingly, our goal is not to compare commercial products against one another, but to benchmark open-source methods against commercial references across multiple data sets, comprising thousands of p*K*_a_ values. This approach also reflects the fact that the training set composition and potential data leakage can be controlled only for the open-source predictors.

Information on the monoprotic, amphoteric, and polyprotic benchmark data sets is found in Table 3, along with similarity values to the combined set of molecules used for fine-tuning in any of the open-source models. Comparison of the three benchmark data sets to the combined fine-tuning data set on a structural basis reveals that they provide equivalent and balanced coverage of roughly the same chemical space (Figure 5). The self-similarity values of the benchmark data sets (defined as the average value of each molecule’s highest similarity to any other molecule in the same data set) and fine-tuning set similarity values are comparable to the values we got for the SAMPL data sets reported in Supplementary Table S3. Distribution of p*K*_a_ values, molecular weights, and others is provided in Supplementary Figure S1.

**Table 3:** Summaries of the Monoprotic, Amphoteric, and Polyprotic Benchmark Datasets with Self-Similarity Values and Similarity Values for the Combined Fine-Tuning Dataset for All of the Methods

evaluation set	number of p*K*_a_ values	number of molecules	self-similarity of the data set	combined fine-tune set similarity
monoprotic set	11,780	11,780	0.557	0.380
amphoteric set	1854	927	0.514	0.390
polyprotic set	4669	2025	0.553	0.380

**Figure 5:**
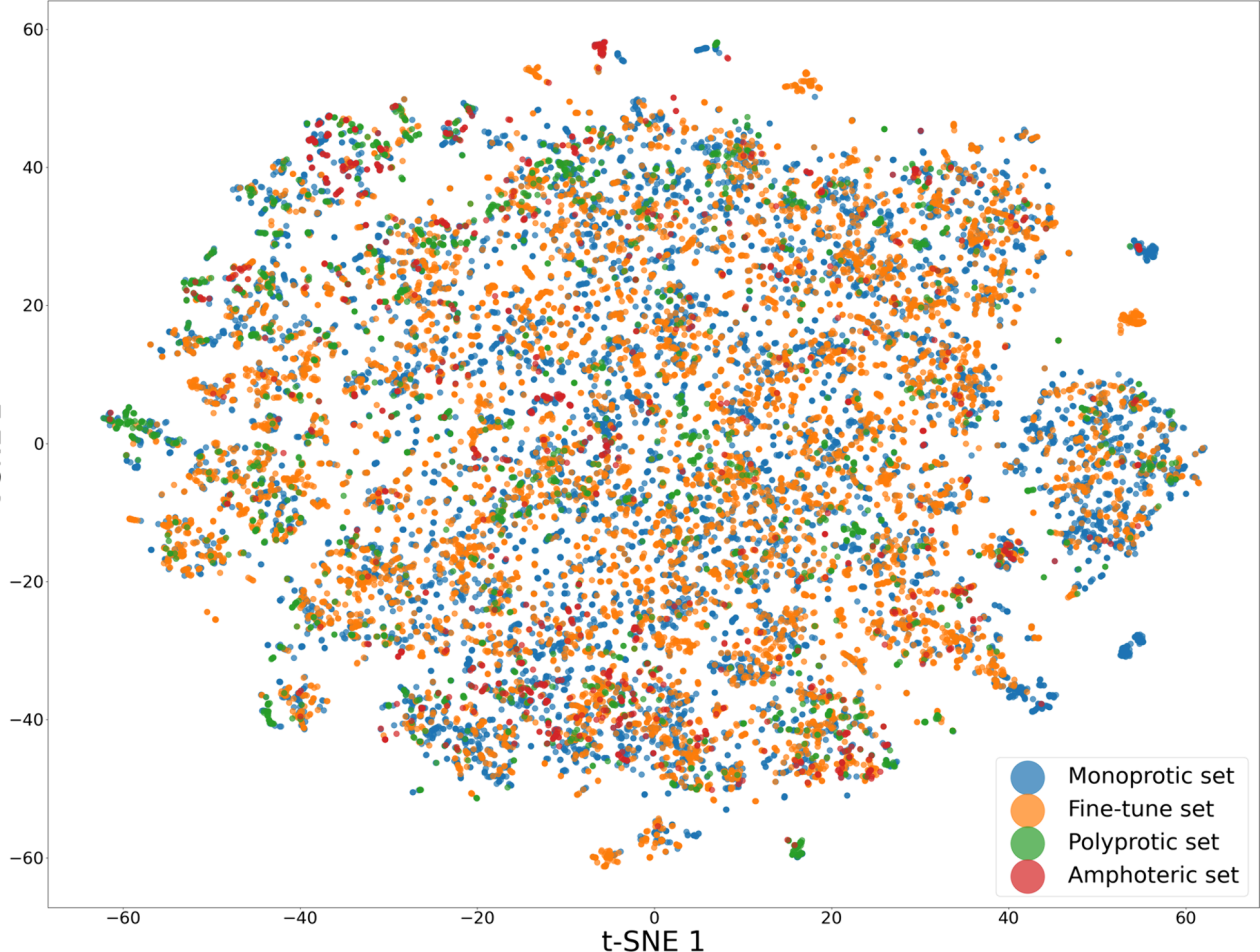
t-SNE plot of the monoprotic, amphoteric, and polyprotic data sets along with the combined fine-tuning data set used by the open-source predictors.

The micro-p*K*_a_ predictors were tested against the monoprotic and the amphoteric benchmark data sets. For the monoprotic set, as there was only one p*K*_a_ value to assign per molecule, we matched the predicted value, which was the closest to the experimental value. For the amphoteric sets, we used a simple linear assignment algorithm to assign those two predictions which provided the lowest sum of absolute deviations. Graphical illustrations of the results along with the distribution of the absolute errors are provided in Figure 6, and the results are also presented in tabular format in Supplementary Table S4.

**Figure 6:**
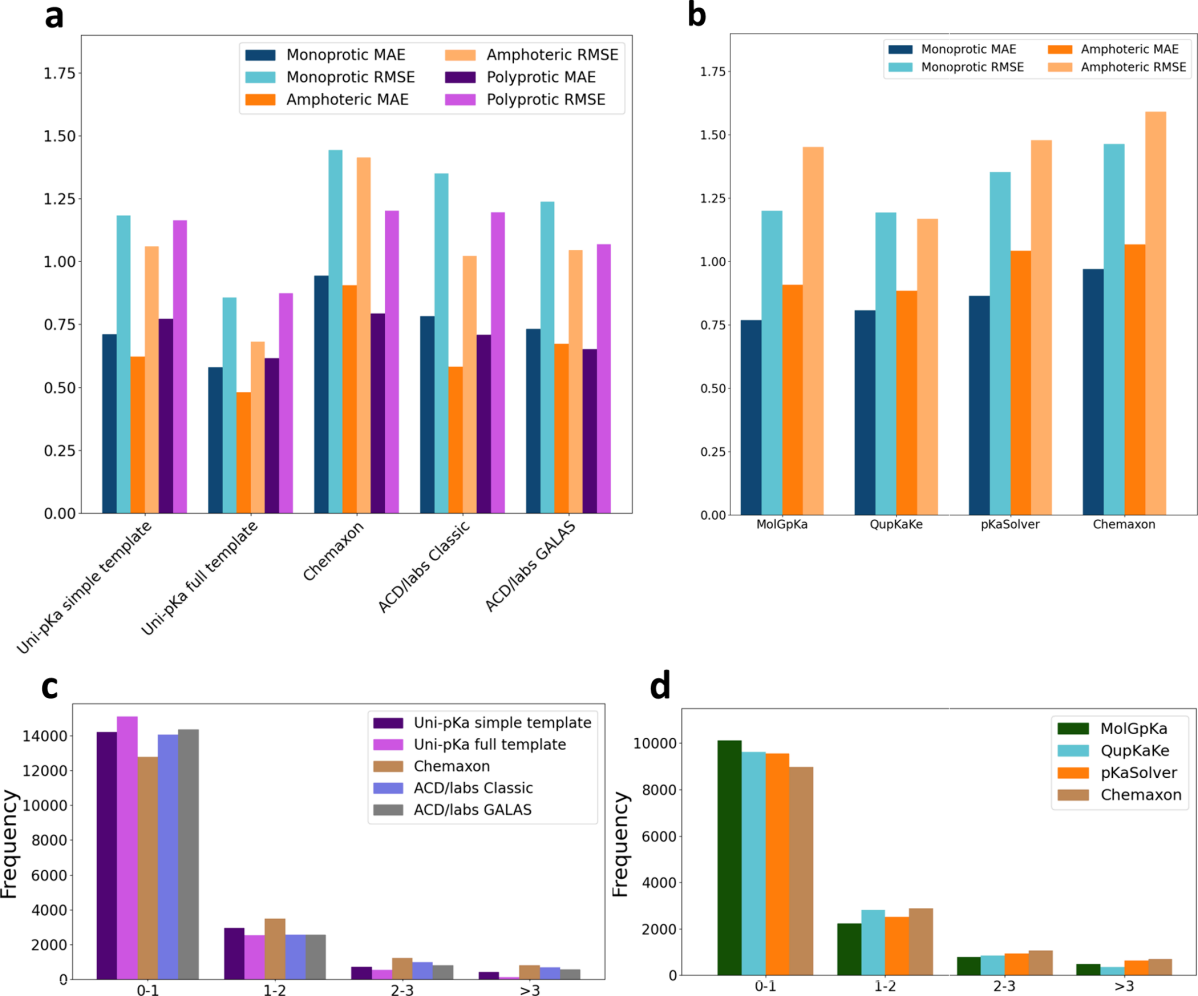
Mean absolute error (MAE) and root mean squared error (RMSE) distributions for the macro p*K*_a_ predictors on the monoprotic, amphoteric, and polyprotic benchmark sets (a) and for the micro p*K*_a_ predictors on the monoprotic and amphoteric benchmark sets (b). Distribution of the number of absolute prediction errors in the given bins (left closed, right open intervals) for the macro p*K*_a_ predictors (c) and the micro p*K*_a_ predictors (d).

The macro-p*K*_a_ predictors were tested against all three benchmark data sets, including the polyprotic set. The matching algorithm was the order-preserving, error-sum-minimizing matching that was also used for charge state transition assignment during database processing. Results are presented in Figure 6, and are available in tabular format in Supplementary Table S5. (The performance of Epik could be positively biased, as this algorithm was used in the preparation of the whole database: we therefore leave it out of the head-to-head comparison here but include its performance metrics for reference in Supplementary Table S6.)

To establish statistical significance between the distribution of prediction errors of different macro and micro-p*K*_a_ predictors, we performed one-way ANOVA, followed by a majority vote of five independent post hoc statistical tests (Supplementary Figure S3). At a significance level of *p* = 0.05, the distribution of the prediction errors of most micro-p*K*_a_ predictors (all except QupKake and MolGpKa) and also most macro-p*K*_a_ predictors (except Uni-p*K*_a_ simple vs ACD/Labs Classic and GALAS) are significantly different.

We can see that open-source predictors have comparable and sometimes significantly better performance than some of the commercial ones by the MAE and RMSE metrics. In general, macro-p*K*_a_ predictors usually outperform micro-p*K*_a_ predictors: this can be due to the more consistent p*K*_a_ description of these models with the nature of experimental results. It is also interesting that QupKake significantly outperforms the Chemaxon predictions, as this model was pretrained on Chemaxon-calculated p*K*_a_ values for molecules in ChEMBL. The simple template enumeration of Uni-p*K*_a_ returns comparable (and, in two cases, significantly better) performances compared to commercial predictors. Altogether, in terms of numerical accuracy, open-source methods, and especially Uni-p*K*_a_, have caught up with commercial products. Reassuringly, the mean absolute errors rarely exceed one p*K*_a_ unit for any of the predictors on any of the data sets. By contrast, commercial providers have a clear edge in exception rates (percentage of compounds that cannot be processed): over the 31,250 compounds of the full database, typically less than 1% threw exceptions on commercial software, while all open-source predictors have shown >1% exception rates, going as high as 6.6% (Uni-p*K*_a_ with the simple template) and 7.4% (pKaSolver).

For drug design purposes, prediction errors within 1 p*K*_a_ unit are typically acceptable, although they can be more problematic as the p*K*_a_ value becomes closer to the physiological pH of 7.4. Here, a larger prediction error could translate to a mischaracterization of the most relevant microspecies (a standard step in ligand preparation), which could propagate to downstream molecular modeling tasks such as solubility or permeability prediction, or docking/virtual screening (via a failed interpretation of protein–ligand interactions). For the latter, typical examples are GPCR ligands, where a protonated amine is usually required to establish a key salt bridge with the D^3.32^ residue, with the p*K*_a_ values of some typical groups being close to 7.4, e.g. 7.9 for the piperazine group of cariprazine, a D2/D3 receptor agonist.^55,56^ (In a broader sense, the specific protonation states at physiologically relevant conditions affect several aspects of drug action, like efficacy, pharmacokinetics, all the way to in vivo pharmacology, and safety.) Larger errors for micro-p*K*_a_ predictors can mostly be attributed to molecules with multiple titratable groups (causing problems due to the incomplete description of the ionization processes by micro-p*K*_a_ predictors). For macro-p*K*_a_ predictors, large errors can be attributed to exotic structures such as strongly lipophilic molecules, macrocycles, conjugated systems, and unusual ionization centers. Some examples of strong outliers (absolute error >3 for at least two predictors) for the macro-p*K*_a_ predictors are provided in Supplementary Figure S4.

### pKaHub: Online Database for the Combined, Annotated Data Set

We uploaded the combined database to a user-friendly web server, accessible at http://pkahub.ttk.hu, with all source code shared on GitHub (https://github.com/keserulab/pkahub). The server implements suitable Django data models for macroscopic charge states and transitions, as well as molecules and microspecies, and stores them along with the curated experimental data, which can be accessed through a graphical interface (as illustrated in Figure 7) or downloaded in tabular, JSON, SMILES, or SDF formats.

**Figure 7:**
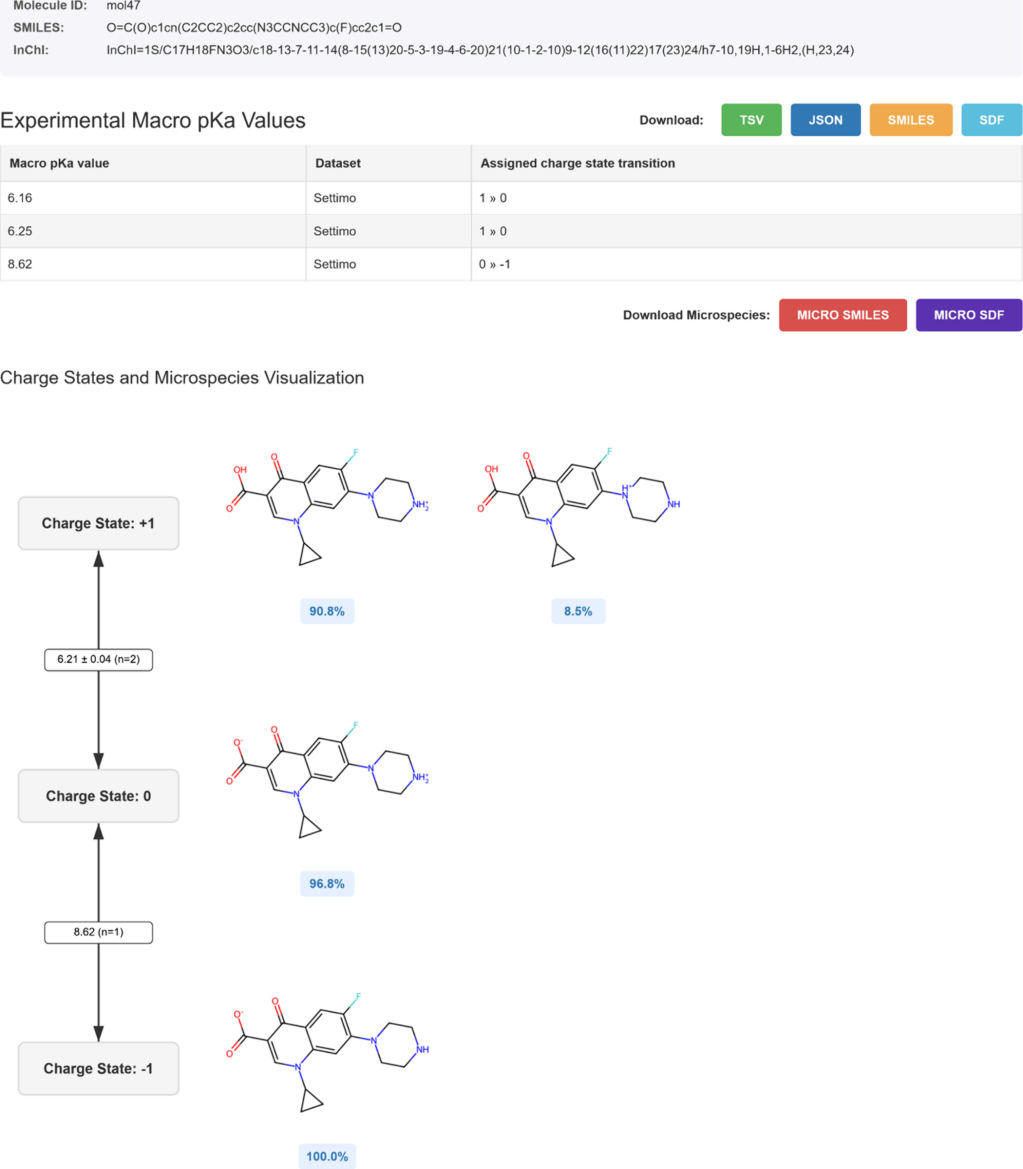
For a given molecule, the user can examine and download the microspecies distribution along with the dissociation constants annotated to macroscopic charge state transitions.

## Conclusion

We compiled a large collection of experimental p*K*_a_ values from the literature and various public resources spanning over 31,000 molecules. The data were carefully curated and filtered: most importantly, this entailed the assignment of experimental p*K*_a_ values to macroscopic charge state transitions through a nonrestrictive, order-preserving matching algorithm. This large corpus of data allowed the thorough testing of four commercial and four open-source predictor models against three data sets with increasingly complex molecules, each with over a thousand data points that were not overlapping with the training data of any of the predictors. The evaluation proved that open-source p*K*_a_ predictors are viable alternatives to commercial ones in terms of the numeric accuracy of the results.

The full curated database is made available for public use on the pKaHub web server (http://pkahub.ttk.hu). The database is annotated according to the modern microequilibria theory of ionization states.

## Supplementary Material



## Data Availability

Molecular structures and experimental p*K*_a_ data, collected from a wide selection of public and literature sources and annotated with macroscopic charge states as described in this paper, are made available for viewing one-by-one or downloading in bulk in multiple formats at the pKaHub web server (http://pkahub.ttk.hu), developed as part of this work. The source code for the web server is provided via GitHub (https://github.com/keserulab/pkahub).

## Notes

Molecule counts over source data sets (Table S1), titratable group definitions and occurrences (Table S2), similarity values (Table S3), property distributions (Figure S1), distribution of experimental errors (Figure S2), performance metrics (Tables S4–S6), ANOVA results (Figure S3), and prediction outliers (Figure S4) (PDF)
