# Supplementary material for: Benchmarking pK a Prediction Algorithms against an Extensive, Public Data Set
Source: J Chem Inf Model. 2026 Apr 6;66(8):4607–19. doi: 10.1021/acs.jcim.6c00107 (PMC13126629; doi:10.1021/acs.jcim.6c00107)
Supplement: Supplementary file 1 [file ci6c00107_si_001.pdf]

# Supplementary information

## Benchmarking p$K_a$ prediction algorithms against an extensive, public dataset

Levente Sipos-Szabó (0009-0002-1210-7940),[1,2] Dávid Bajusz (0000-0003-4277-9481),[1,*] György T. Balogh (0000-0001-8273-1760),[3,4,5] György M. Keserű (0000-0003-1039-7809)[1,2]

*[1] Medicinal Chemistry Research Group and Drug Innovation Centre, HUN-REN Research Centre for Natural Sciences, Magyar tudósok krt. 2, 1117 Budapest, Hungary*

*[2] Department of Organic Chemistry and Technology, Faculty of Chemical Technology and Biotechnology, Budapest University of Technology and Economics, Műegyetem rkp. 3., H-1111 Budapest, Hungary*

*[3] Department of Pharmaceutical Chemistry, Semmelweis University, Hőgyes, Endre u. 9, Budapest, 1092, Hungary*

*[4] Center for Pharmacology and Drug Research & Development, Semmelweis University, Üllői út 28, Budapest, 1085, Hungary*

*[5] Department of Chemical and Environmental Process Engineering, Faculty of Chemical Technology and Biotechnology, Budapest University of Technology and Economics, Műegyetem rkp. 3., H-1111 Budapest, Hungary*

*Corresponding author email: bajusz.david@ttk.hu

**Table of contents**

Table S1: Molecule counts over source datasets ........ S2
Table S2: Titratable group definitions and occurrences ........ S2
Table S3: Similarity values ........ S4
Figure S1: Property distributions ........ S5
Figure S2: Distribution of experimental errors ........ S6
Tables S4-6: Performance metrics ........ S6
Figure S3: ANOVA results ........ S8
Figure S4: Prediction outliers ........ S9

**Supplementary Table S1.** Number of source dataset-specific molecules and the fraction of these molecules per source dataset. (A molecule is dataset-specific if it only appears in that given dataset.)

*Organic Oxygen Acids and Nitrogen Bases

| Source Dataset Name | Total Number of Molecules | Number of source dataset-specific molecules | Fraction of source dataset-specific molecules |
|---|---|---|---|
| Settimo | 426 | 24 | 0.0563 |
| Hunt | 2242 | 432 | 0.1931 |
| Jensen | 48 | 11 | 0.2292 |
| Caine | 71 | 63 | 0.8873 |
| Manchester | 85 | 83 | 0.9765 |
| Novartis | 280 | 267 | 0.9536 |
| AvLiLuMoVe | 123 | 4 | 0.0325 |
| OOAs and NBs* | 1123 | 46 | 0.041 |
| Datawarrior | 6375 | 409 | 0.0676 |
| IUPAC digitized | 10615 | 2750 | 0.3362 |
| AttenGpKa training set | 15712 | 4345 | 0.4184 |
| OCHEM | 11855 | 1334 | 0.1201 |
| QSARToolbox | 13390 | 4662 | 0.3778 |
| Baltruschat Chembl | 6303 | 4108 | 0.6742 |
| SAMPL6 | 24 | 22 | 0.9167 |
| SAMPL7 | 20 | 3 | 0.15 |
| SAMPL8 | 21 | 19 | 0.9048 |
| euroSAMPL1 | 35 | 34 | 0.9714 |

**Supplementary Table S2**. Investigated titratable functional groups/substructures with their SMARTS pattern, acid/base labeling, functional group classes and the number of molecules in the database which contains the given substructure.

| **Substructure** | **SMARTS** | **Acid or base** | **Class** | **Molecule count** |
|---|---|---|---|---|
| Sulfate monoether | [SX4:0](=[O:1])(=[O:2])(-[O:3])-[OX2:4]-[H:5] | A | Sulfur acids | 20 |
| Sulfonic acid | [SX4:0](=[O:1])(=[O:2])(-[#6,#7:3])-[OX2:4]-[H:5] | A | Sulfur acids | 642 |
| Sulfinic_acid | [SX3:1](=[O:2])-[O:3]-[H] | A | Sulfur acids | 28 |
| Thiosulfuric acid | [S:0]~[SX4:1](~[O:2])(~[O:3])-[O:4]-[H:5] | A | Sulfur acids | 1 |
| Carbo(di)thioic acid | [CX3;!$(C(=O)[OX2H]):0](=[O,S:1])-[OX2H,SX2H:2] | A | Sulfur acids | 71 |
| Phosphate | [PX4:1](=[O:2])(-[OX2:3]-[H])(-[O+0:4])-[OX2:5]-[H] | A | Phosphorous acids | 226 |

| | | | | |
|---|---|---|---|---|
| Phosphate diester | [PX4:1](=[O:2])(-[OX2H0:3]-[*:4])(-[OX2H0:5]-[*:6])-[OX2H:7] | A | Phosphorous acids | 131 |
| Phosphonate | [PX4:1](=[O:2])(-[OX2:3]-[H])(-[C,c,N,n:4])-[OX2:5]-[H] | A | Phosphorous acids | 351 |
| Phosphonate ester | [PX4:1](=[O:2])(-[OX2:3]-[C,c,N,n,F,Cl,Br,I:4])(-[C,c,N,n,F,Cl,Br,I:5])-[OX2:6]-[H] | A | Phosphorous acids | 71 |
| Phosphinic acid | [PX4:1](=[O:2])(-[C,c,N,n,F,Cl,Br,I:3])(-[C,c,N,n,F,Cl,Br,I:4])-[OX2:5]-[H] | A | Phosphorous acids | 60 |
| Carboxyl | [C:1](=[O:2])-[O:3]-[H] | A | Carbon acids | 6511 |
| Carboxyl acid enol | [C:0]=[C:1](-[OX2:2]-[H:3])-[OX2:4]-[H:5] | A | Carbon acids | 10 |
| Hydroxy-enone | [O:1]=[C;R:2]-[C;R:3]=[C;R:4]-[O:5]-[H] | A | Carbon acids | 197 |
| Phenol | [c,n,o:1]-[O:2]-[H] | A | Phenol | 4735 |
| Pheny thiol | [c,n:1]-[SX2:2]-[H] | A | Thiol/Phenyl thiol | 227 |
| Thiol | [C,N:1]-[SX2:2]-[H] | A | Thiol/Phenyl thiol | 325 |
| Peroxide | [OX2:1]-[O:2]-[H] | A | Other | 81 |
| N-hydroxyamide | [C:1](=[O:2])-[N:3]-[O:4]-[H] | A | Hydroxilamine | 307 |
| Hydroxylamine | [C,c:1]-[O:2]-[NH2:3] | A | Hydroxilamine | 23 |
| N-hydroxylamine | [NH:1]-[O:2]-[H] | A | Hydroxilamine | 259 |
| Oxime | [$([#7]:,=[#6,#7]),$([#7]:,=[#6,#7]:,-[#6,#7]:,=[#6,#7]):0]-[OX2,NX3:1]-[H:2] | A | Oxime | 1333 |
| Amide | [C:1](=[O:2])-[N:3]-[H] | A | Amide/Imide | 4662 |
| Imide cyclic | [CX3;R:1](=[O,S:2])-[NX3;H1;R:3]-[CX3;R:4](=[O,S:5]) | A | Amide/Imide | 368 |
| Imide acyclic | [CX3;!R:1](=[O,S:2])-[NX3;H1;!R:3]-[CX3;!R:4](=[O,S:5]) | A | Amide/Imide | 51 |
| Sulfonamide | [SX4:1](=[O:2])(=[O:3])-[NX3+0:4]-[H] | A | Sulfonamide/Phosphamide | 1702 |
| Phosphamide | [PX4:0](=[O:1])-[NX3:2]-[H:3] | A | Sulfonamide/Phosphamide | 83 |
| Seleninic acid | [SeX3:0](=[O:1])(-[#6,#7:2])-[OX2:3]-[H:4] | A | Other | 26 |
| Selenenic acid | [SeX2:0]-[OX2:1]-[H:2] | A | Other | 4 |
| Selenol | [SeX2:0]-[H:1] | A | Other | 13 |
| Arsenic acid | [AsX4:0](=[O:1])(-[#6,#7:2])-[OX2:3]-[H:4] | A | Other | 74 |

| | | | | |
|---|---|---|---|---|
| Hydrazine | [NX3,NX4+:1]-[NX3,NX4+:2] | B | Hydrazine | 519 |
| Primary amine | [NX3:0](-[CX4,c,$(C=C):1])(-[H])-[H] | B | Primary amine | 5019 |
| Secondary amine | [NX3:0](-[CX4,c,$(C=C):1])(-[CX4,c,$(C=C):2])-[H] | B | Secondary amine | 4535 |
| Tertiary amine | [NX3:0](-[CX4,c,$(C=C):1])(-[CX4,c,$(C=C):2])(-[CX4,c,$(C=C):3]) | B | Tertiary amine | 5848 |
| Imine | [#6,#7,P,S:0]=[NX2:1] | B | Imines | 3988 |
| Basic aromatic nitrogen | [n;H0;X2:1] | B | Basic aromatic nitrogen | 10275 |
| Pyrrolic nitrogen | [n:1]-[H] | A | Pyrrolic nitrogen | 2759 |

**Supplementary Table S3.** Self-similarity values and similarity values compared to the combined fine-tuning set for the benchmark datasets used for evaluations and the SAMPL datasets as reference.

| Dataset name | Self-similarity | Combined fine-tune set similarity |
|---|---|---|
| **Monoprotic set** | 0.557 | 0.380 |
| **Amphoteric set** | 0.514 | 0.390 |
| **Polyprotic set** | 0.553 | 0.380 |
| **SAMPL6** | 0.594 | 0.385 |
| **SAMPL7** | 0.593 | 0.334 |
| **SAMPL8** | 0.466 | 0.350 |
| **euroSAMPL1** | 0.214 | 0.356 |

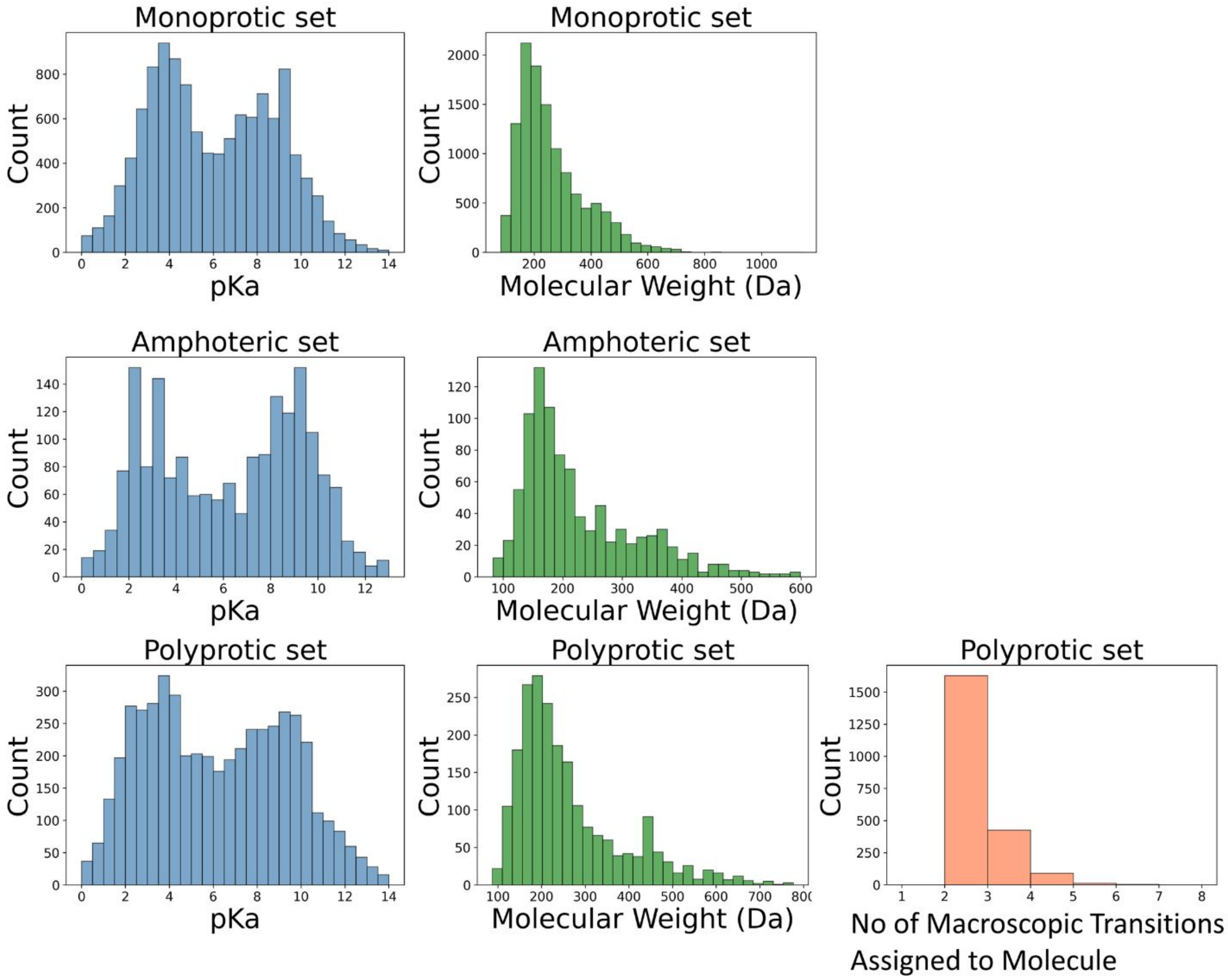


**Supplementary Figure S1.** Distribution of pKa, molecular weight and macroscopic charge state transition per molecule for the datasets used for predictor evaluation.

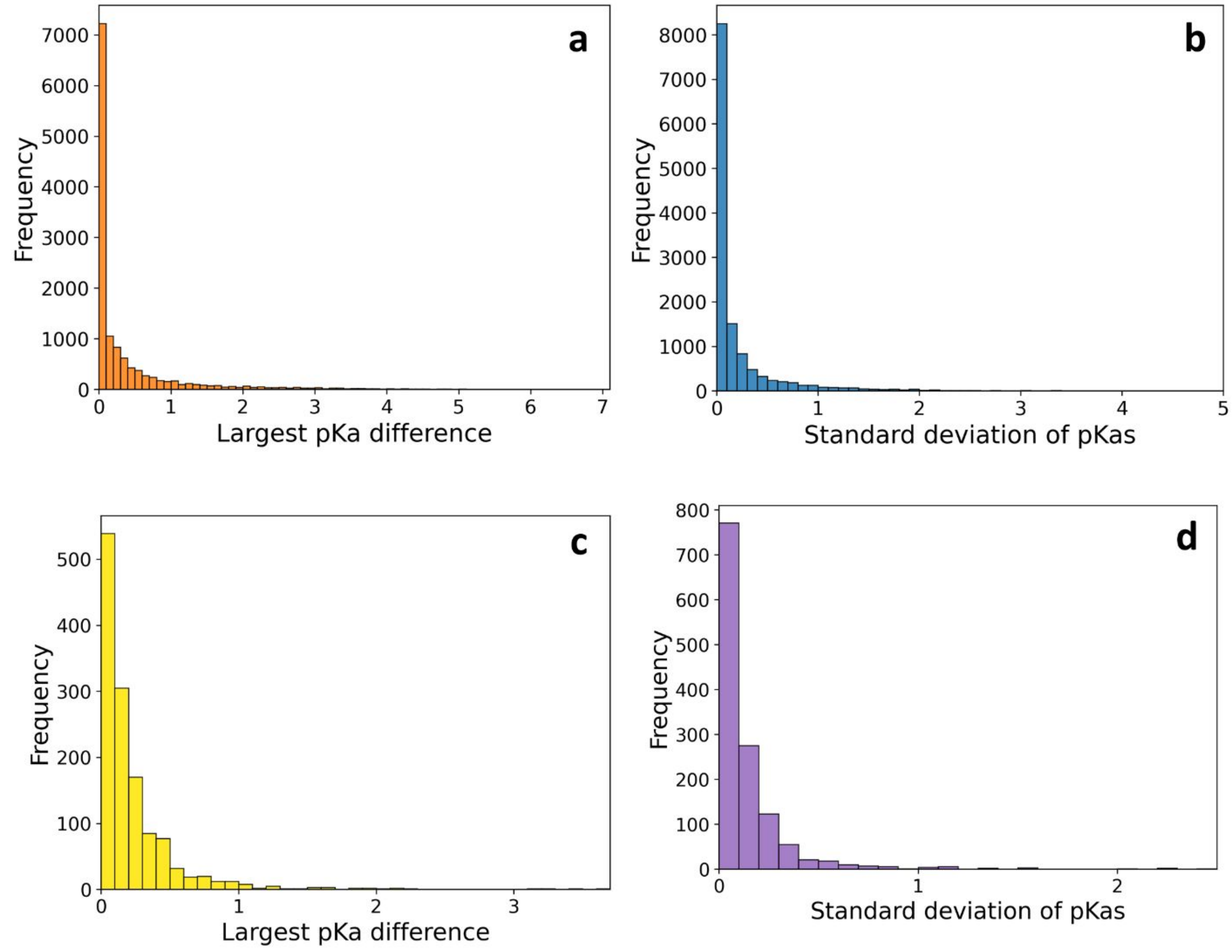


**Supplementary Figure S2.** Distribution of largest pKa differences between two experiments for the same molecule and charge state transition *for the whole dataset* (a). Distribution of the standard deviations of pKa values from different experiments for the same molecule and charge state transition *for the whole dataset* (b). *Distribution of* largest pKa differences between two experiments for the same molecule and charge state transition *for the IUPAC digitized pKa dataset (c).* Distribution of standard deviation of pKa values from different experiments for the same molecule and charge state transition *for the IUPAC digitized pKa dataset* (*d*).

**Supplementary Table S4.** Performance expressed as mean absolute errors (MAE) and root mean squared errors (RMSE) for the micro-p$K_a$ predictors on the Monoprotic and Amphoteric datasets

| **Micro p$K_a$ predictor** | **Performance metric** | **Monoprotic set** | **Amphoteric set** |
|---|---|---|---|
| **MolGpKa** | **MAE** | 0.770 | 0.909 |
| | **RMSE** | 1.200 | 1.452 |
| **pKaSolver** | **MAE** | 0.865 | 1.042 |
| | **RMSE** | 1.353 | 1.479 |

| | | | |
|---|---|---|---|
| **QupKake** | **MAE** | 0.807 | 0.885 |
| | **RMSE** | 1.194 | 1.168 |
| **Chemaxon** | **MAE** | 0.970 | 1.067 |
| | **RMSE** | 1.463 | 1.591 |

**Supplementary Table S5.** Performance expressed as mean absolute errors (MAE) and root mean squared errors (RMSE) for the macro-p$K_a$ predictors on the Monoprotic, Amphoteric and Polyprotic datasets.

| **Macro p$K_a$ predictor** | **Performance metric** | **Monoprotic set** | **Amphoteric set** | **Polyprotic set** |
|---|---|---|---|---|
| **Uni-p$K_a$ simple template** | **MAE** | 0.711 | 0.623 | 0.772 |
| | **RMSE** | 1.183 | 1.060 | 1.164 |
| **Uni-p$K_a$ full template** | **MAE** | 0.579 | 0.480 | 0.616 |
| | **RMSE** | 0.856 | 0.682 | 0.873 |
| **Chemaxon** | **MAE** | 0.943 | 0.906 | 0.792 |
| | **RMSE** | 1.442 | 1.414 | 1.202 |
| **ACD/labs Classic** | **MAE** | 0.783 | 0.582 | 0.709 |
| | **RMSE** | 1.350 | 1.021 | 1.195 |
| **ACD/labs GALAS** | **MAE** | 0.731 | 0.672 | 0.651 |
| | **RMSE** | 1.237 | 1.044 | 1.069 |

**Supplementary Table S6.** Performance expressed as mean absolute errors (MAE) and root mean squared errors (RMSE) for Epik on the Monoprotic, Amphoteric and Polyprotic dataset.

| **Micro p$K_a$ predictor** | **Performance metric** | **Monoprotic set** | **Amphoteric set** | **Polyprotic set** |
|---|---|---|---|---|
| **Epik** | **MAE** | 0.613 | 0.586 | 0.622 |
| | **RMSE** | 0.926 | 0.841 | 0.909 |

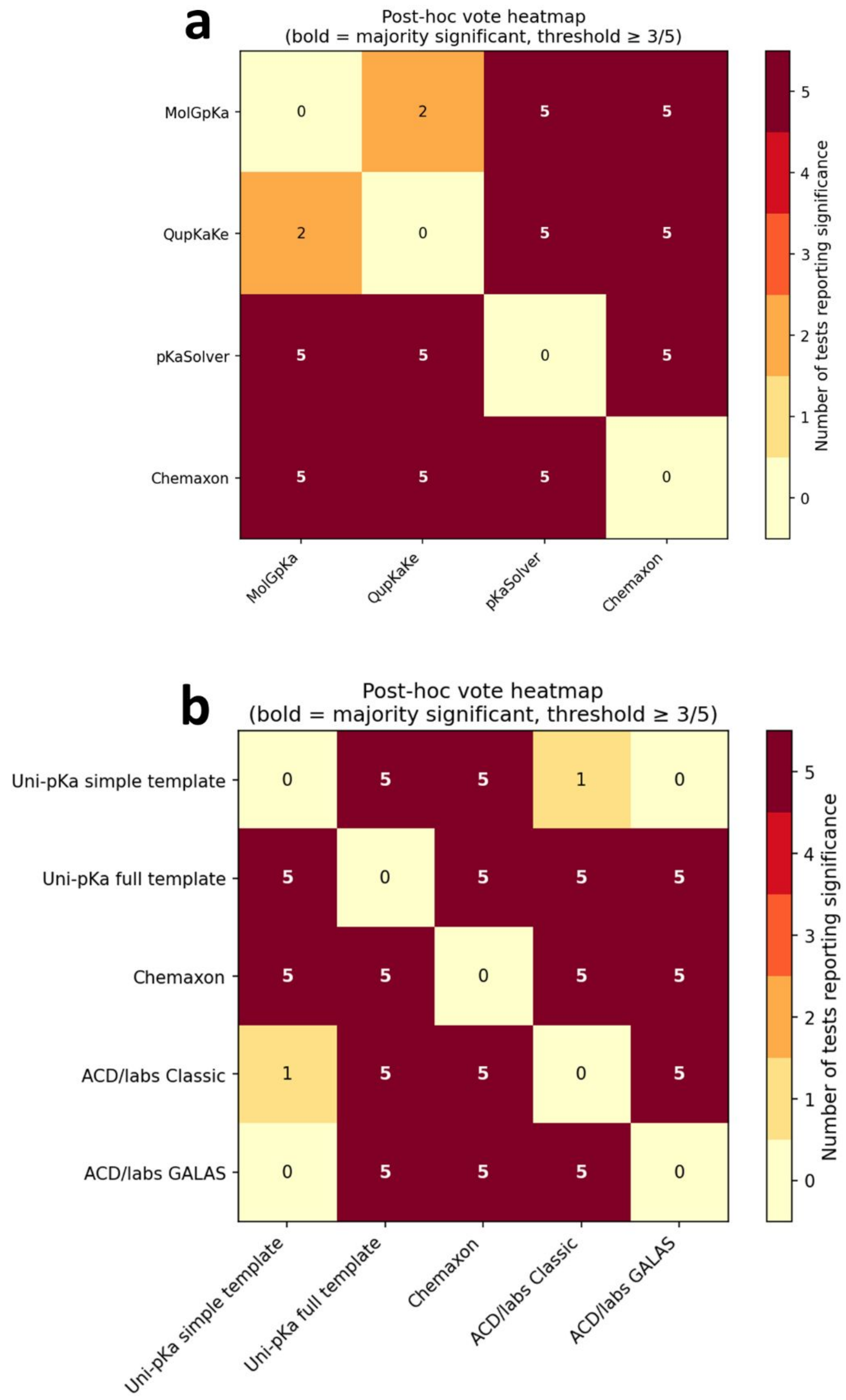


**Supplementary Figure S3.** Pairwise statistical comparison of absolute prediction errors across a) four micro p$K_a$ predictors and b) five macro p$K_a$ predictors. A one-way analysis of variance (ANOVA) confirmed that at least one predictor pair differed significantly in absolute prediction error. Post-hoc pairwise

comparisons were performed using five independent parametric tests. The heatmap displays, for each predictor pair, the number of tests (out of five) that reported a statistically significant difference. Pairs for which a majority of tests (≥ 3/5) reached significance are indicated in bold; all remaining pairs are shown in regular weight. The colour scale ranges from white (0 tests significant) to dark red (all 5 tests significant). Diagonal entries are zero by definition.

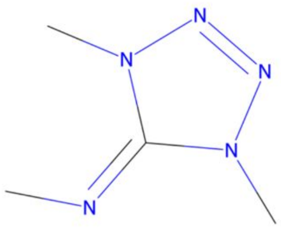

| Exp pKa | Uni-pKa ST | Uni-pKa FT | Chemaxon | ACD/labs Classic | ACD/labs GALAS |
|---|---|---|---|---|---|
| 9.57 | 4.55 | 4.55 | 6.70 | 10.80 | 14.19 |

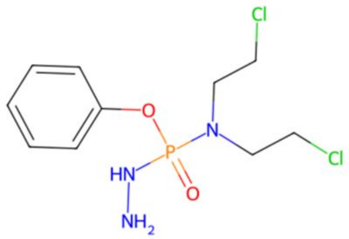

| Exp pKa | Uni-pKa ST | Uni-pKa FT | Chemaxon | ACD/labs Classic | ACD/labs GALAS |
|---|---|---|---|---|---|
| 10.46 | 10.46 | 10.46 | 13.53 | 5.89 | 2.33 |

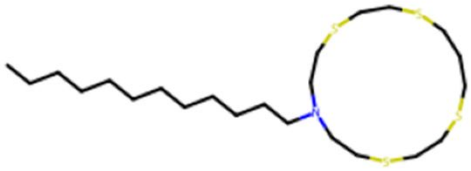

| Exp pKa | Uni-pKa ST | Uni-pKa FT | Chemaxon | ACD/labs Classic | ACD/labs GALAS |
|---|---|---|---|---|---|
| 3.81 | 6.32 | 6.32 | 10.74 | 7.78 | 8.14 |

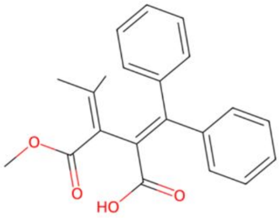

| Exp pKa | Uni-pKa ST | Uni-pKa FT | Chemaxon | ACD/labs Classic | ACD/labs GALAS |
|---|---|---|---|---|---|
| 6.33 | 3.59 | 3.59 | 3.56 | 3.48 | 3.46 |

**Supplementary Figure S4.** Structure of outlier molecules (absolute pKa prediction error > 3 for at least two predictors) for the macro pKa predictors.
